# A Clinical Audit on the Monitoring and Management of Antipsychotic-Induced Hyper Prolactinaemia

**DOI:** 10.1192/bjo.2024.590

**Published:** 2024-08-01

**Authors:** Noman Khan

**Affiliations:** South West Yorkshire Foundation Trust, Yorkshire, United Kingdom

## Abstract

**Aims:**

Antipsychotic medications are one of the major iatrogenic causes of hyperprolactinaemia with the attendant short- and long-term effects and risks associated with it.

- The audit sought to answer the question: Are we monitoring and managing hyper prolactinemia caused by anti psychotic medications appropriately?

**Methods:**

- A literature search for relevant data and standards with regards to monitoring and management of hyperprolactinaemia was conducted.
- The audit was based on the standards derived from South West Yorkshire NHS Partnership Foundation Trust’s (SWYPFT) standards, NICE guidelines, and the Maudsley Prescribing Guidelines in Psychiatry (14th edition), focusing on the Trust's standards.
- The total population under consideration included every patient under the care of the North Kirklees, Community mental health team (CMHT), Old age psychiatry services (OPS) that was using antipsychotic medication in the time period between 16 June 2022 and 15th July 2023.

**Results:**

- Total patients 61
- Female 30
- Male 31
- Age: 65 and above
- Already on antipsychotic: 49
- Started on antipsychotic: 12
- Two or more antipsychotic: 2
- Switch from one antipsychotic to other: 4
- Prolactin monitoring not required: 27 because were already using olanzapine, quetiapine and aripiprazole

**Monitoring required: 34**

- Initiation: 12
- Prolactin level done 2/12
- Prolactin level not done 10/12
- LAI (long acting antipsychotic) 11
  Prolactin level done 7/11
  Prolactin level not done 4/12
  Not done: 75% were with Care coordinator
  25% Wellbeing team
- On oral antipsychotic that require prolactin level monitoring: 11
- Prolactin level done 5/11
- Prolactin level not done 6/11

**Conclusion:**

Patients who were on antipsychotics in community required prolactin monitoring. In more than 50% of patients prolactin were not monitored regularly because of communication gap between Psychiatrist and GPs as no clear instructions were mentioned from Psychiatrist to GPs, patients and care coordinators.

A small number of patients in whom prolactin was raised were highlighted to their respective medics and managed accordingly.

